# Evolutionary Double-Bind Treatment Using Radiation Therapy and Natural Killer Cell-Based Immunotherapy in Prostate Cancer

**DOI:** 10.1016/j.ijrobp.2025.09.034

**Published:** 2025-09-23

**Authors:** Kimberly A. Luddy, Jeffrey West, Mark Robertson-Tessi, Bina Desai, Andrew Ojeda, Hannah Newman, Veronica Estrella, Taylor M. Bursell, Sarah Barrett, Jacintha O’Sullivan, Laure Marignol, Robert A. Gatenby, Joel S. Brown, Alexander R.A. Anderson, Cliona O’Farrelly

**Affiliations:** a Department of Cancer Biology and Evolution, Moffitt Cancer Center, Tampa, Florida; b Integrated Mathematical Oncology Department, Moffitt Cancer Center, Tampa, Florida; c Trinity St. James’s Cancer Institute, Radiobiology and Molecular Oncology Research Group, Applied Radiation Therapy Trinity, Discipline of Radiation Therapy, Trinity College Dublin, Dublin, Ireland; d School of Biochemistry and Immunology, Trinity Biomedical Sciences Institute, Trinity College Dublin, Dublin, Ireland

## Abstract

**Purpose:**

Evolution-informed therapies exploit evolutionary consequences of drug resistance to inhibit treatment resistance and prolong time to progression. One strategy, termed an evolutionary double-bind, uses an initial therapy to elicit a specific adaptive response by cancer cells, which is then selectively targeted by a follow-on therapy. Although the concept of an evolutionary double-bind has long been hypothesized in cancer, it has not been measured. Here, to our knowledge, we present the first example of a quantifiable double-bind: radiation therapy (RT) with natural killer (NK) cells. RT induces lethal double-strand DNA breaks, but cancer cells adapt. Although this increases resistance to DNA-damaging agents, it also enhances expression of NK cell ligands creating an obvious choice for a double-bind strategy.

**Methods and Materials:**

We investigated this potential evolutionary double-bind through in vitro studies and evolution-based mathematical models. Using multiple prostate cancer cell lines, we evaluated surface and soluble NK ligand expression following RT. In vitro competition experiments were performed with an isogenic radiation-resistant cell line model. We introduced a two-population Lotka-Volterra competition model, consisting of radiation-sensitive and radiation-resistant populations modeling intrinsic growth rates with fixed carrying capacity and inter-specific competition terms.

**Results:**

Alterations in NK cell ligands resulted in a twofold increase in sensitivity to NK cell-mediated killing with selective targeting of RT-resistant cells. These dynamics were framed mathematically to quantify the double bind. RT alone slowed overall growth but strongly selected for RT-resistant cells. NK cell therapy alone suppressed the RT-resistant population, but with a surviving population of radiation-sensitive cells. Model simulation predicted that optimal tumor control would be achieved through initial RT followed by NK cells. Subsequent experiments confirmed the model prediction.

**Conclusions:**

We conclude that RT and NK cell-based immunotherapy produce an evolutionary double-bind. This multidimensional approach addresses the immediate challenge of treatment resistance and lays the groundwork for the development of personalized treatment regimens tailored to the evolving dynamics of individual tumors.

**Significance:**

Clinical experience demonstrates that prostate cancer has a remarkable capacity to evolve resistance to all currently available treatments resulting in progression and, ultimately, patient death. Resistance mechanisms often come at a fitness cost placing cells in a bind when competing with surrounding cells. A carefully chosen secondary drug can introduce a double-bind targeting the adaptive resistance mechanism. This manuscript provides the first direct experimental evidence quantifying an “evolutionary double-bind’ in prostate cancer supporting the combination of DNA-damaging agents and NK cell-based immunotherapy in evolutionarily guided treatment designs. Our work is mathematically novel in that it extends Evolutionary Game Theory models and establishes an experimental-mathematical framework to quantify genuine evolutionary double binds applicable across cancer types and treatment modalities.

## Introduction

In the USA, deaths from prostate cancer (PC) totaled 34,700 in 2023 compared with 27,540 in 2015.^1^ Thus, despite the introduction of several new life-prolonging therapies, mortality rates continue to rise.^2^ Clinical experience has demonstrated that PC cells have a remarkable capacity to evolve resistance to all currently available treatments resulting in tumor progression and, ultimately, patient death.^3,4^ It is, therefore, not exaggerating to assert that evolution is the proximate cause of death in PC.

Evolution-informed therapies are novel therapeutic strategies with the following 2 key objectives: (1) reduce overall tumor burden while also (2) preventing the outgrowth of a fully resistant phenotype.^5–7^ Therapy resistance represents a fundamental barrier to curative treatment strategies. Resistance occurs when a population of tumor cells contains preexisting traits or acquires adaptive traits enabling their survival during therapy. Evolution-informed strategies often exploit the fitness cost of this resistance. That is, the investment of cancer cell resources to produce, maintain, and operate the molecular machinery of resistance increases fitness when treatment is administered but decreases fitness in the absence of treatment.^8,9^ The latter typically results in a proliferative disadvantage compared with nonresistant cells in the absence of treatment.^8,10^ Furthermore, the molecular machinery itself can produce an increased sensitivity to a second treatment or alter the tumor cell’s interactions with the immune system.^11^ These dynamics can be exploited by generating an “evolutionary double bind” in which the mechanism(s) of resistance to the first therapy produce specific vulnerabilities that can be targeted by a subsequent treatment.^10,12,13^

**Fig. 1.**
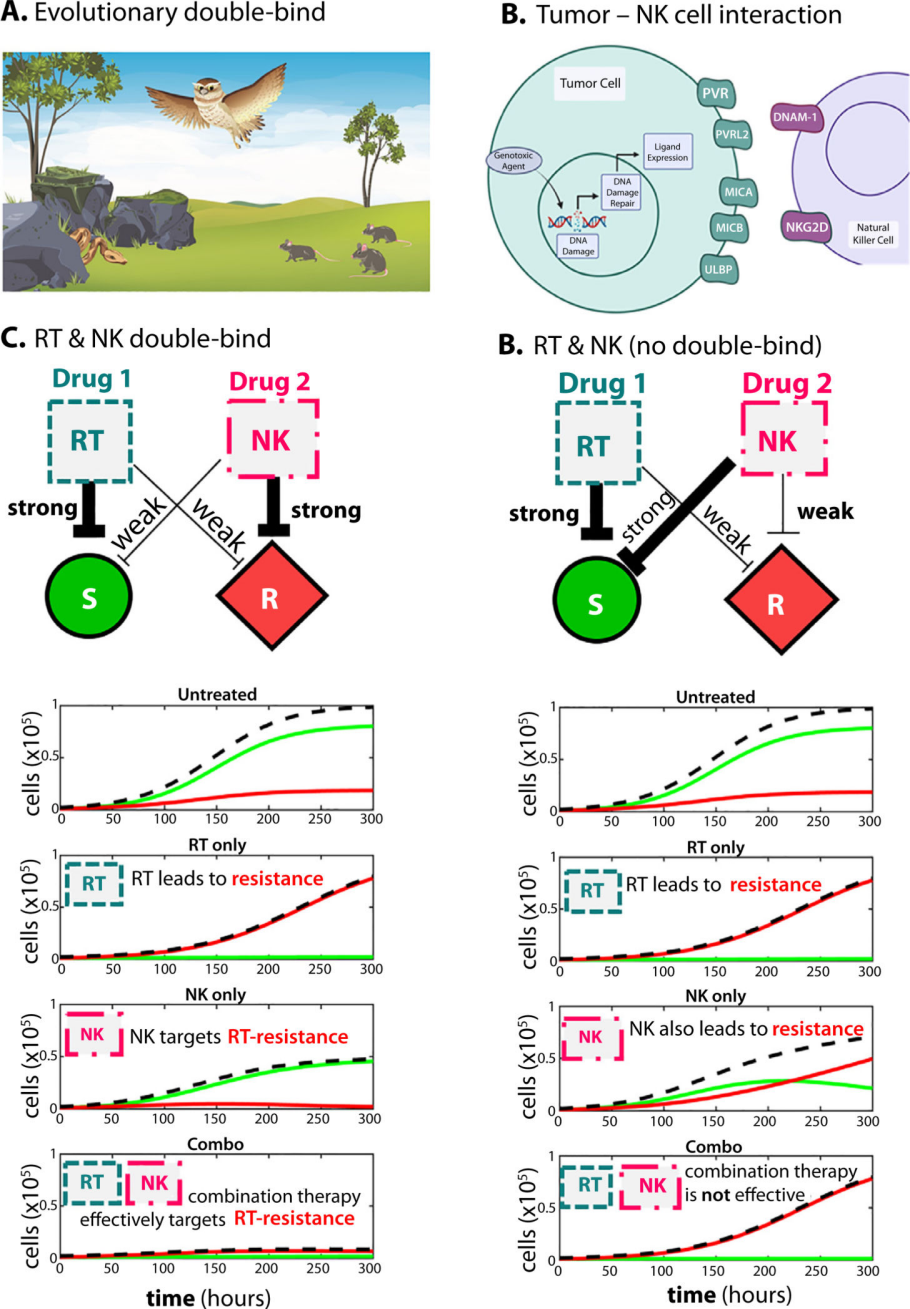
Evolutionary double-bind therapy uses 2 therapies that explicitly target populations with different modes of therapy resistance. (A) The owl-snake dynamic is used to illustrate the ecological concept of an evolutionary double bind. (B) NK cell ligands are expressed in response to genotoxic stressors including radiation therapy (RT). (C) Hypothesized double-bind between RT and natural killer (NK) cells. RT strongly targets RT-sensitive cells (green) and weakly targets resistant cells (red). NK cells strongly target RT-resistant cells and weakly target RT-sensitive. Example treatment trajectories are shown, in which RT leads to RT-resistance, NK leads to RT-sensitivity, and the combination effectively targets both cell types. (D) In contrast, without a double-bind, NK cells may strongly target RT-sensitive cells, rendering NK and combination therapy not effective in eliminating RT-resistant cells.

The evolutionary double-bind strategy for treating cancer was derived from “predator facilitation” seen with owls and snakes.^12,14^ Dessert rodents adapt to predation from owls by hiding. This increases the hunting efficiency of desert snakes that reside in the brush (Fig. 1A).^14^ A double bind in cancer occurs when the first therapy facilitates the efficacy of the second therapy. Importantly, because of these dynamics, the second therapy may be highly effective in the context of a double bind but much less so when given as an initial treatment.

Radiation therapy (RT) and immunotherapy are an appealing combination for cancer treatment.^15,16^ Given locally, RT induces double-stranded DNA breaks in tumor cells initiating cellular stress responses that trigger either cell death or DNA repair and cell survival.^17^ T cell-based immunotherapies benefit from RT-induced activation of the local immune response and novel tumor antigens released during RT that can generate a systemic immune reaction.^15,18,19^ RT also changes the protein expression on surviving cancer cells. Most notably, it has been shown that RT, as well as other DNA-damaging agents, increases natural killer (NK) cell ligand expression on tumor cells (Fig. 1B).^20,21^

NK cells induce apoptosis in cancer cells with perforins and granzymes packed into cytolytic granules, and through the release of death-inducing ligands such as Fas ligand and TNF-related apoptosis-inducing ligand.^22,23^ Additionally, activated NK cells support the adaptive immune response through the release of chemokines and cytokines including interferon gamma, TNF, granulocyte-macrophage colony-stimulating factor, interleukin, CCL3, CCL4, and CCL5.^24^ The activation of NK cells relies on a combination of inhibitory and activating signals received from target cells.^25,26^ Activation of NK cells occurs only when these signals result in a net activation signal, that is, when activating proteins on the surface of NK cells achieve a larger signal than regulatory proteins. Tumor cells often increase inhibitory signals (eg, altering ligand expression on tumor cells) as an adaptive strategy for immune evasion.^27–29^

Here, we hypothesize that the altered expression of NK ligands on tumor cells in a RT-resistant population would increase the sensitivity to NK cell-mediated killing, creating an opportunity for an evolutionary double-bind treatment strategy. In a true double-bind (left column, Fig. 1C), NK cell-based therapy would select for radiation-sensitive cells (green). When radiation is applied, selection for RT resistance (red) occurs. As seen in the schematic, this requires that NK therapy strongly inhibits radiation-resistant cells (red, R) while weakly inhibiting RT-sensitive cells (green, S). This criterion results in the best response for combination therapy: inhibiting the growth of both sensitive and resistant cells (Fig. 1C). Untreated conditions lead to a growth of both RT-sensitive (green) and RT-resistant (red) cells, in which RT-sensitive cells outcompete RT-resistant cells due to a small cost of resistance.

By contrast, the right column illustrates the scenario when the criterion for a double-bind is not met (Fig. 1D). Here, NK cell-based therapy strongly inhibits radiation-sensitive cells and only weakly inhibits RT-resistant cells. Under combination therapy, radiation resistance is still under strong positive selection, illustrating the lack of an evolutionary double-bind without the strong-weak inhibition criterion.

The success of double-bind therapy will depend on the properties of the tumor and the treatments used. Finding optimal double-bind strategies (eg, when to start each treatment, duration, dose) requires calibration of both cell-intrinsic characteristics (eg, growth rates, costs of resistance) and cell-extrinsic interaction dynamics (eg, cell competition) within the heterogeneous mix of tumor cell phenotypes being therapeutically targeted. To study these complex dynamics and identify optimal strategies, we developed mathematical models to describe the key cell-intrinsic and cell-extrinsic features of radiation-sensitive, radiation-resistant, and NK cells. Mathematical modeling, including approaches such as partial differential equations,^30^ spatial agent-based models,^31^ in silico clinical trials,^32^ and ordinary differential equations,^33^ is a commonly used approach to test the efficacy of immunotherapy treatment schedules to mitigate resistance and assess the combination of RT with immunotherapy.^34–36^

We calibrated the mathematical models using in vitro experiments. Here, the cost of resistance is defined as a reduction in the growth rate of the resistant line when compared with its treatment-naïve counterpart. Interaction dynamics (eg, cooperation or competition between cell types) were quantified using Evolutionary Game Theory (EGT) mathematical models parameterized from in vitro competition assays of treatment-naïve isogenic lines in co-culture with RT-resistant lines. Preferential targeting was quantified by extending these competition assays to include NK cells. Taken together, results from our in vitro model demonstrate the potential for combining RT and NK cells as an evolutionarily informed treatment strategy in advanced PC.

## Results

### Radiation increases expression of NK receptor ligands

**Fig. 2.**
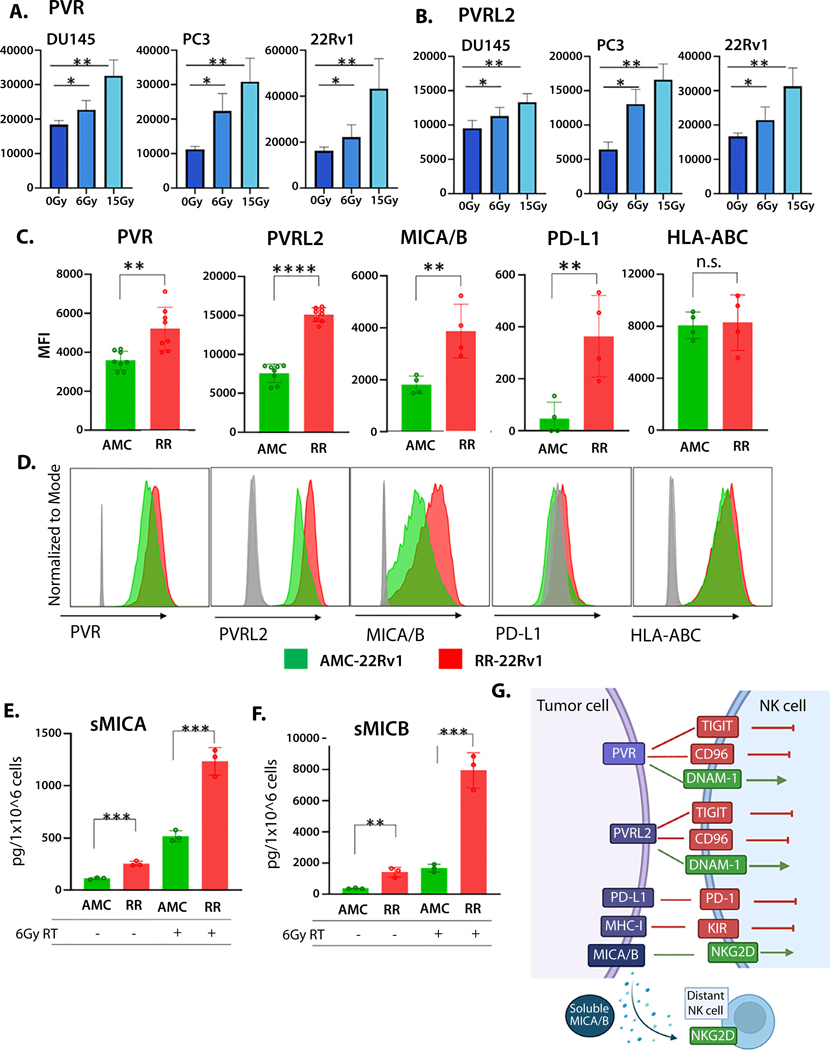
NK ligand expression in prostate cancer. Surface expression of PVR (A) and PVRL2 (B) by prostate cancer cell lines 48 hours after radiation with 0, 6, or 15 Gy. Constitutive expression of NK ligands measured in an isogenic model of RT-resistant prostate cancer. Radiation-sensitive (AMC-22Rv1, green) and radiation-resistant 22Rv1 (RR-22Rv1, red) cell lines measured by flow cytometry, mean fluorescence intensity (MFI) (C), and representative histograms (isotype [gray], AMC [green], RR [red]) (D). Soluble MICA (E) and soluble MICB (F) were measured by enzyme-linked immunosorbent assays. Illustration of the effects of NK receptor ligation on NK cell function (G). n.s. = not significant, *P < .05, **P < .01, ***P < .001, ****P < .0001

Radiation increases expression of multiple NK ligands and immune costimulatory and inhibitory markers in a variety of cell types. Here, we investigated changes in NK ligands, poliovirus receptor (PVR), and poliovirus receptor-related 2 (PVRL2, CD112), on human PC cell lines, DU145, PC3, and 22Rv1 after radiation. Surface expression was significantly increased in all 3 cell lines 48 hours after treatment (0, 6, or 15 Gy) (Fig. 2A, B).

An isogenic cell line model of RT resistance was previously developed using thirty 2 Gy fractionated doses (total 60 Gy) on 22 Rv1 PC cells as demonstrated in supplemental Figure 1A, B and previously published.^37^ Here, we measured the surface expression of NK receptor ligands, PVR, PVRL2 (CD112), and MHC class I polypeptide-related sequences A and B (MICA/B) on control (AMC-22Rv1) and radiation-resistant (RR-22Rv1) cells. PVR and PVRL2 bind activating NK receptor DNAX accessory molecule 1 (DNAM-1, CD226) or inactivating NK receptors T cell immunoreceptor with Ig and ITIM domains (TIGIT) and CD96 (Fig. 2G). We found that expression of PVR and PVRL2 was significantly increased on RR-22Rv1 (Fig. 2C, D) (*P* < .01 and *P* < .0001, respectively). Natural killer group 2 member D (NKG2D) ligands, MICA and MICB, were upregulated on radiation-resistant cells compared with control (Fig. 2C, D) (*P* < .01). The mean fluorescence intensity (MFI) for checkpoint ligand programmed death-ligand 1 (PD-L1) increased significantly in the radiation-resistant cell line (*P* < .01) (Fig. 2C, D). However, both lines had very low levels (<400 MFI) of PDL-1 expression. The biological relevance of these low levels remains unknown. Human leukocyte antigen ABC (HLA-ABC, MHC class I) is often upregulated following RT; however, constitutive HLA-ABC was not altered in our isogenic cell line model (Fig. 2C, D).

Surface expression of MICA and MICB on tumor cells activates NK cells through binding with NKG2D (Fig. 2G). Exterior portions of MICA and MICB can be proteolytically cleaved by several metalloproteases resulting in soluble MICA/B proteins (sMICA, sMICB) that maintain full ligand function (Fig. 2G). Using enzyme-linked immunosorbent assays, we measured the amount of cleaved MICA and MICB in the media of control and radiation-resistant cells before and after radiation. RR-22Rv1 cells have higher levels of sMICA (Fig. 2E) and sMICB (Fig. 2F) than control lines. Radiation increased levels in both cell lines (Fig. 2E, F).

### Radiation-resistant cells are more sensitive to NK cell-mediated killing

**Fig. 3.**
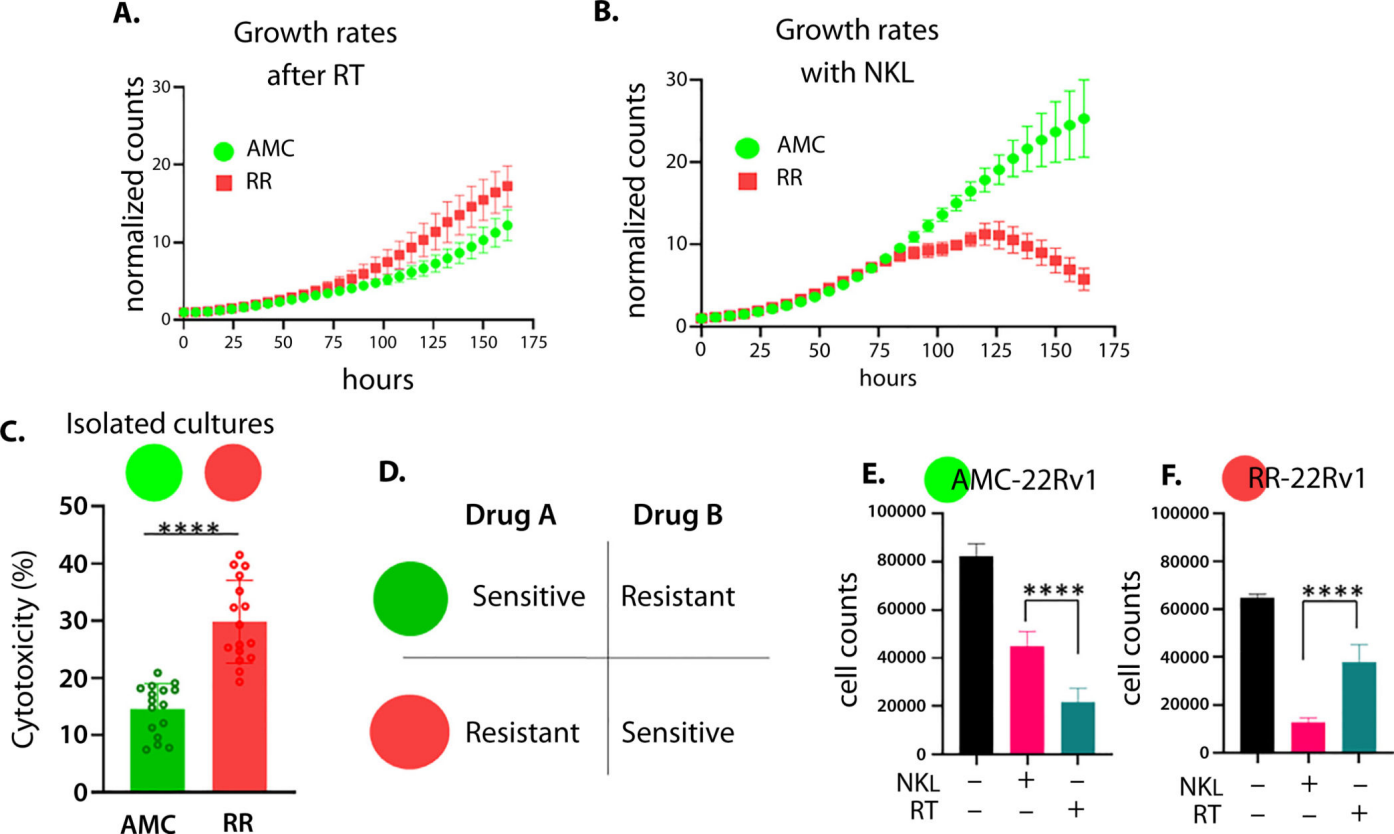
Radiation-resistant prostate cancer cells are more sensitive to NK cell-mediated killing. Live imaging assays of isolated cultures of radiation-sensitive cells (AMC, green) and radiation-resistant cells (RR, red) following radiation (6 Gy) (A) and during challenge with NKL cells (5:1 NK:target cell ratio) (B). Radiation-resistant cells (RR) (red) were more sensitive to killing by NKL in a 24-hour flow-based assay (10:1 NK:target cell ratio) (C). Evolutionary double-bind occurs when 1 treatment targets 1 population and the second treatment preferentially targets another population (D). Cell counts using IncuCyte imager after 7 days of treatment with either 6 Gy RT (blue bar) or NKL cells (pink bar) demonstrate that the radiation-sensitive population is inhibited by RT more than NK cells (E) and that radiation-resistant cells are inhibited by NK cells more than RT (F). n.s. = not significant, **P < .01, ***P < .001, and ****P < .0001.

NK cell activation occurs when activation signals exceed inhibitory signals from target cells (Fig. 2G). Increased NK ligand expression on tumor cells can increase sensitivity to NK cell-mediated killing; however, many NK receptor ligands bind activating and inhibitory receptors on NK cells. Therefore, we directly measured sensitivity to NK cells in our cell lines. AMC-22Rv1 cells and RR-22Rv1 cells were plated in separate wells and treated with RT (6 Gy) or co-cultured with an immortalized NK cell line, NKL. Figure 3 illustrates the potential evolutionary relationship between RT-resistance and NK cell killing. Under RT, RR cells showed outpaced growth compared with AMC-22Rv1 cells (Fig. 3A). By contrast, increased sensitivity to NK cells by the radiation-resistant line was seen using live imaging (IncuCyte, 5:1 E:T ratio) (Fig. 3B) and in short-term assays using flow cytometry (24 hours, 10:1 E:T) (Fig. 3C). A similar trend was observed at various effector to target ratios (Fig. E2A, B).

An evolutionary double bind requires differential sensitivity to 2 treatments. One treatment must strongly select against sensitive tumor cells, whereas the second treatment strongly selects against cells resistant to the first. Therefore, when the second treatment is applied, cells that were initially sensitive to the first will be selected for, and cells resistant to the first will be selected against (Fig. 3D). To determine if NK cells represent an evolutionary double bind with RT in our isogenic cell lines, we compare sensitivity of each line to each treatment (Fig. 3E, F). AMC-22Rv1 cells are more sensitive to RT than to NK cells (Fig. 3E), whereas RR-22Rv1 cells are more sensitive to NK cells than to RT (Fig. 3F).

### Outcomes are dependent on initial seeding frequency in vitro

**Fig. 4.**
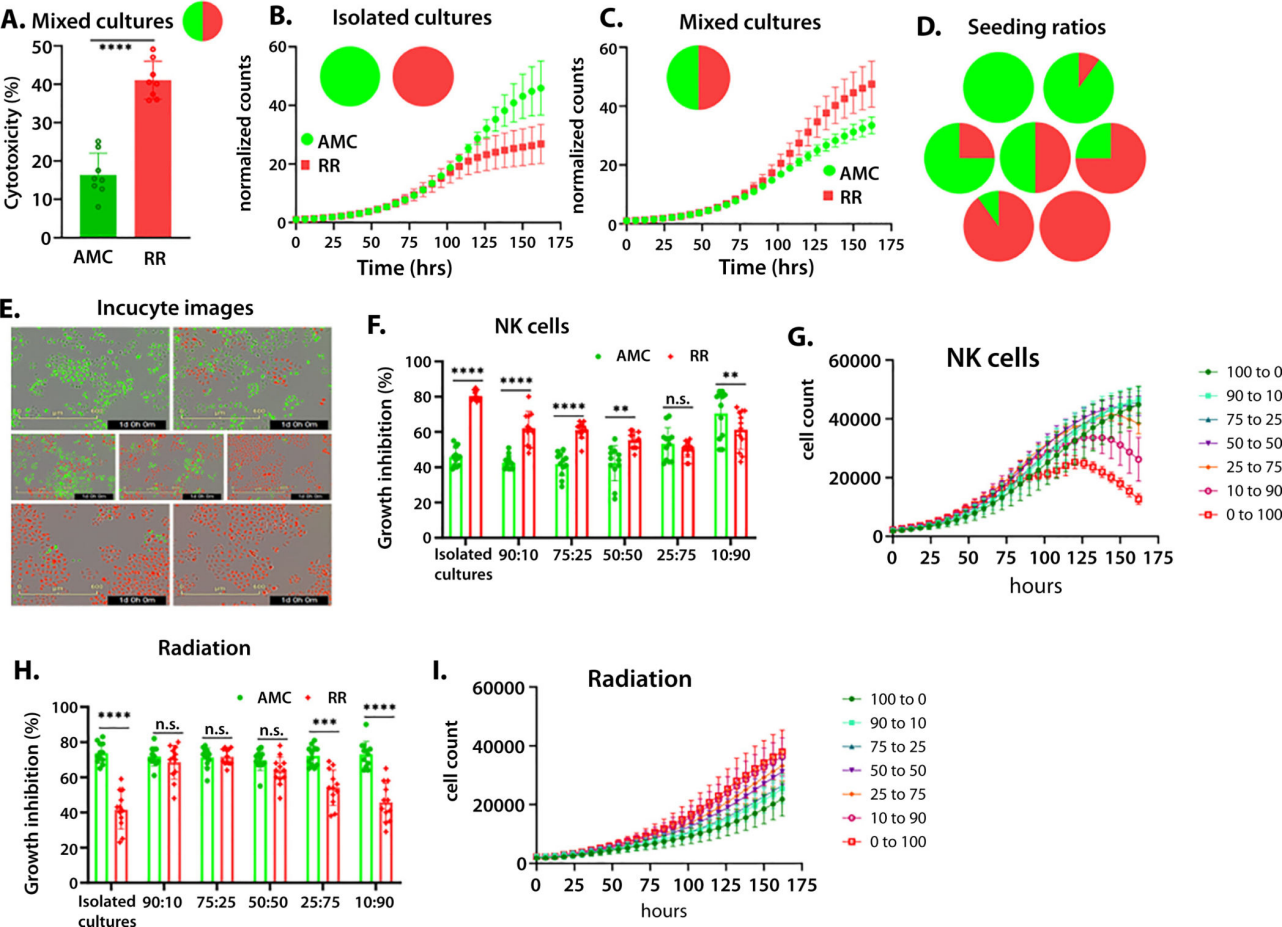
Treatment sensitivity is dependent on initial seeding frequency in mixed cultures. NKL cells (10:1 effector: target ratio) preferentially target radiation-resistant (RR) cells in mixed cultures (24 hours, 50:50 AMC:RR) (A). AMC and RR cell lines have differential growth dynamics when cultured in isolated wells (B) or in mixed cultures (50:50 AMC:RR) (C). Radiation-sensitive (green) and radiation-resistant (red) cells were seeded at various frequencies (0:100, 90:10, 75:25, 50:50, 25:75, 10:90, 0:100 AMC:RR) (D) and imaged over 7 days (E) in the presence of NKL (5:1 effector: target ratio) (F-G) or following radiation (6Gy) (H-I). n.s. = not significant, **P < .01, ***P < .001, and ****P < .0001.

Treatment sensitivity assays are often performed with populations cultured in separate dishes. This ignores interaction effects occurring between 2 populations that may affect growth dynamics and treatment sensitivity required for a double bind. Therefore, we repeated the 24-hour flow-based assays in culture dishes seeded with AMC and RR cells in the same well along with NK cells. The NK cells preferentially targeted the RR cells (Fig. 4A) similar to when the 2 tumor cell lines were challenged with NK cells in isolation (Fig. 3C).

Interestingly, the intrinsic growth rates of the 2 cell lines differ in 50:50 mixed cultures compared with isolated cultures in the absence of treatment (Fig. 4B, C), indicating an interaction effect between the 2 populations, in which RR-22Rv1 cells inhibit the growth of radiation-sensitive cells and AMC-22Rv1 cells facilitate the growth of radiation-resistant cells. To determine the consequence of these interaction effects on the RT/NK double bind, we cultured fluorescently labeled cells (AMC-22Rv1 GFP, RR-22Rv1 mCherry) at various seeding ratios (Fig. 4D, E). We then used live imaging to measure growth rates of the 2 cell lines under selection with NK cells or a single dose of RT. NK cells were most effective when RR-22Rv1 cells were cultured alone or seeded at a high frequency (10:90 AMC:RR) (Fig. 4F, G, Fig. E3A). Higher growth inhibition of RR-22Rv1 by RT occurred in cultures with higher numbers of AMC-22Rv1 cells, and RT was most effective in cultures with a higher frequency of radiation-sensitive cells (Fig. 4H, I, Fig. E3B).

### Two treatments are better than one

**Fig. 5.**
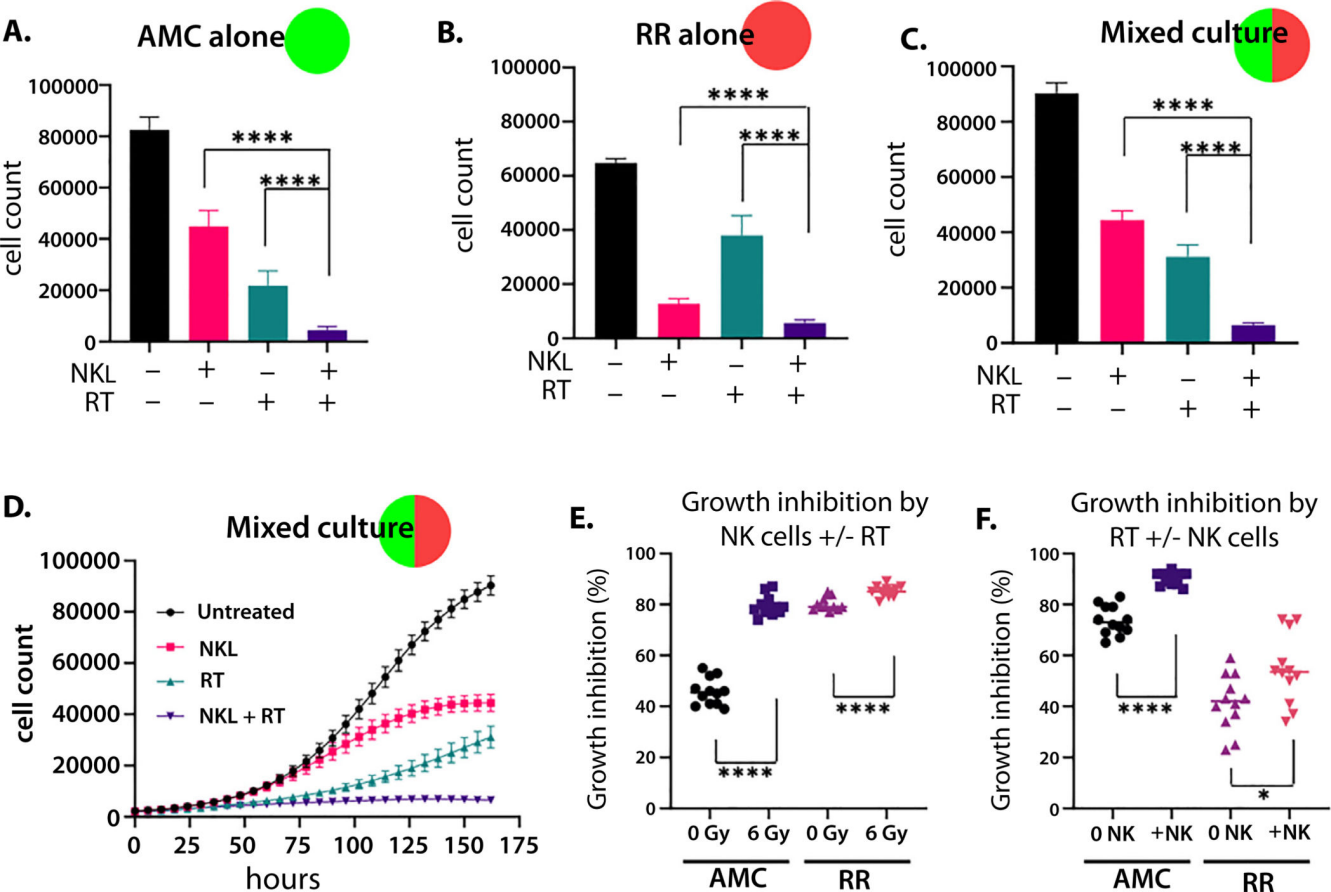
Radiation therapy (RT) and NK cells are most effective in combination. Cell counts using an IncuCyte imager after 7 days of treatment with NKL cells (5:1 effector to target ratio) (pink bars), 6 Gy RT (blue bars), or combination (purple bars) of radiation-sensitive (AMC) cell line (A), the radiation-resistant (RR) cell line (B), and AMC co-cultured with RR, 50:50 AMC:RR (C-D). Growth inhibition by NK cells with and without RT (E). Growth inhibition by RT with and without NK cells (F).

Combination therapy is often more effective than monotherapy. Using our in vitro isogenic cell line model, we irradiated all seeding ratios (Fig. 4D, E) with 6 Gy RT and later added NK cells (5:1 effector to target ratio). Combination therapy with sequential RT followed by NK cells was more effective than either monotherapy in AMC-22Rv1 cells (Fig. 5A) and RR-22Rv1 cells (Fig. 5B) in both mono-cultures and mixed cultures (50:50 ratio) (Fig. 5C, D). Combination therapy had the largest effect on the radiation-sensitive population. Giving RT prior to NK cells significantly increased the sensitivity of the AMC population to NK cells (Fig. 5E), and the addition of NK cells increased the efficacy of RT against the AMC population, although to a lesser degree (Fig. 5F). This effect was observed less in the RR-22Rv1 cells (Fig. 5E, F). Combination therapy was most effective in all seeding ratios (Fig. E4).

### Mathematical modeling quantifies an evolutionary double bind

**Fig. 6.**
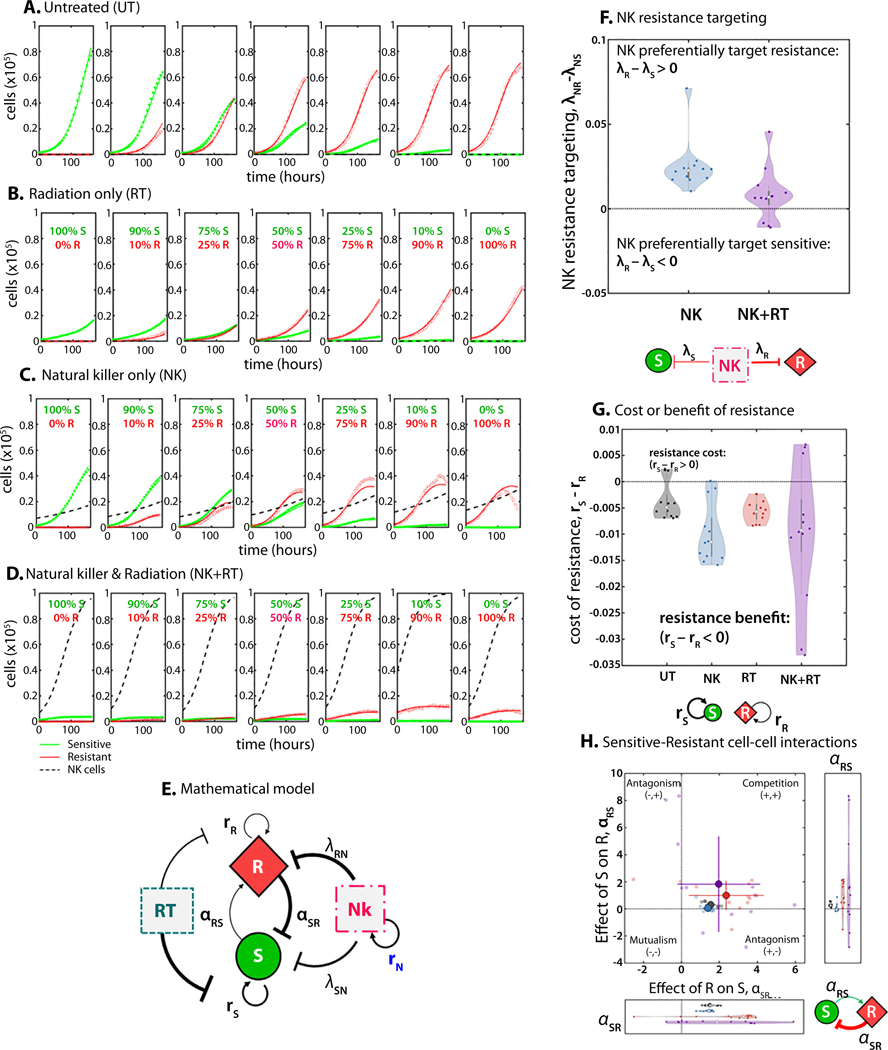
Mathematical model of sensitive and resistant dynamics under therapy. The mathematical model (Eqs. 1–3) was fit across each initial resistant fraction simultaneously and repeated for each of the 12 replicates (see the “Materials and Methods” section) to give a distribution of parameter values, for untreated (A), radiation therapy (RT) (B), natural killer (NK) (C), and RT+NK (D). (E) Interaction network of the mathematical model. (F) When NK cells are present (NK-only or NK+RT), the competitive effect of NK cells on resistant cells (*λ*_NR_) is stronger than the competitive effect of NK cells on sensitive cells (*λ*_NS_), indicative of an evolutionary double-bind. (G) The cost of radiation resistance (*r*_*S*_-*r*_*R*_) is shown to be negative across all treatment conditions, indicating an intrinsic growth benefit in favor of RT-resistant cells. (H) Competition dynamics between RT-sensitive and RT-resistant cells indicate “competitive exclusion,” in which RT-resistant cells outcompete RT-sensitive cells (*λ*_SR_, *λ*_RS_ > 0). The effect of *R* on *S* is much stronger than that of *S* on *R*.

Next, we developed a mathematical model designed to quantify important cell-intrinsic features (eg, cell-line-specific growth rates) and cell−cell interaction dynamics. The mathematical model (Fig. 6E and Fig. E5) is a Lotka-Volterra (LV) competition model between RT-sensitive (AMC) and RT-resistant (RR). LV and EGT mathematical frameworks have been extensively applied to cancer modeling^38^ often paired with experimental data to describe competition^39^ facilitation,^40^ or coexistence^41^ between cancer cell populations, and to investigate the importance of the cost of resistance.^8^ Noncancer cell types (eg, immune cells) can be considered players in the game or alter the parameterization of game dynamics among cancer cell types.^42,43^

This mathematical model accounts for cell-type-dependent growth rates (*r*_*S*_, *r*_*R*_), the competitive effect of cell type *j* on cell type *i* (⍺_*ij*_), and cell-type-dependent carrying capacities (*K*_*S*_, *K*_*R*_). The model also includes NK cells, which grow with logistic growth dynamics and kill sensitive and resistant cells at a rate of λ_*R*_ and λ_*S*_, respectively. The mathematical model was fit to co-culture data (Fig. 6A-D), in which cells are seeded across a range of initial RR fractions: *f* = [0, 0.1, 0.25, 0.50, 0.75, 0.9, 1]. The model was fit to this full range simultaneously and repeated across 12 replicates. Using least-squares error minimization, the model provided good agreement with the data (Fig. 6A-D). The distribution of estimated parameters that provided the best fit across replicates is shown in Table 1.

The mathematical model allowed us to evaluate our hypothesized existence of a double-bind by comparing parameters describing the inhibition of radiation-sensitive (λ_SN_) and radiation-resistant (λ_RN_) cells by NK cells. NK cells preferentially target RR cells (and thus represent a true double bind) if the difference in these inhibition rates is positive, ie, λ_RN_ - λ_SN_ > 0. Figure 6F confirms the existence of the double bind in NK cell therapy alone (blue) and in combination with RT (purple): λ_RN_ - λ_SN_ > 0. This effect is most pronounced with NK therapy alone, but is still present with combination therapy.

### The double bind does not require a cost of resistance

Next, we investigated whether a cost to radiation-resistance exists in terms of growth rate differences between sensitive and resistant populations (*r*_*S*_ > *r*_*R*_). In Figure 6G, model analysis indicates a lack of cost of resistance—indeed, an intrinsic growth benefit is conferred to RR cells across all treatment conditions (*r*_*R*_ > *r*_*S*_). However, this benefit is offset by a lower carrying capacity (see Table 1) such that *K*_*R*_ < *K*_*S*_. Interestingly, it appears that a cost of resistance to the intrinsic growth rate is not a necessary condition for an evolutionary double bind.

Finally, we quantified the competitive effects of RT-sensitive cells on RR cells (⍺_RS_) and the inverse (⍺_SR_). RT-sensitive cells have a weak effect on RR cells (⍺_RS_≈ 0), whereas RR cells strongly suppress RT-sensitive cells (⍺_SR_ > 0). These competition parameters are plotted in Figure 6H to classify interactions between sensitive and resistant populations into 4 possible categories: mutualism, competition, sensitive antagonism, or resistant antagonism. This confirms previous results of competitive dynamics in alternative radiation-sensitive and resistant cell lines.^44^ Again, it appears that a frequency-dependent fitness disadvantage of RR cells is not a necessary condition for an evolutionary double bind.

### Clinical relevance of an evolutionary double bind

Next, to illustrate the clinical relevance of a second drug that fits the definitions of an evolutionary double-bind, we developed a simplified mathematical model with the following components: logistic growth, shared carrying capacity, a cost of resistance, and a double-bind parameter, as seen in Eqs. 4–5 (see the “Materials and Methods” section). The double-bind parameter (*B*) modifies the second drug’s target by either only targeting sensitive cells (*B* = 0), only targeting resistant cells (*B* = 1), or as a ratio of efficacy against sensitive and resistant cells (0 < *B* < 1).

**Fig. 7.**
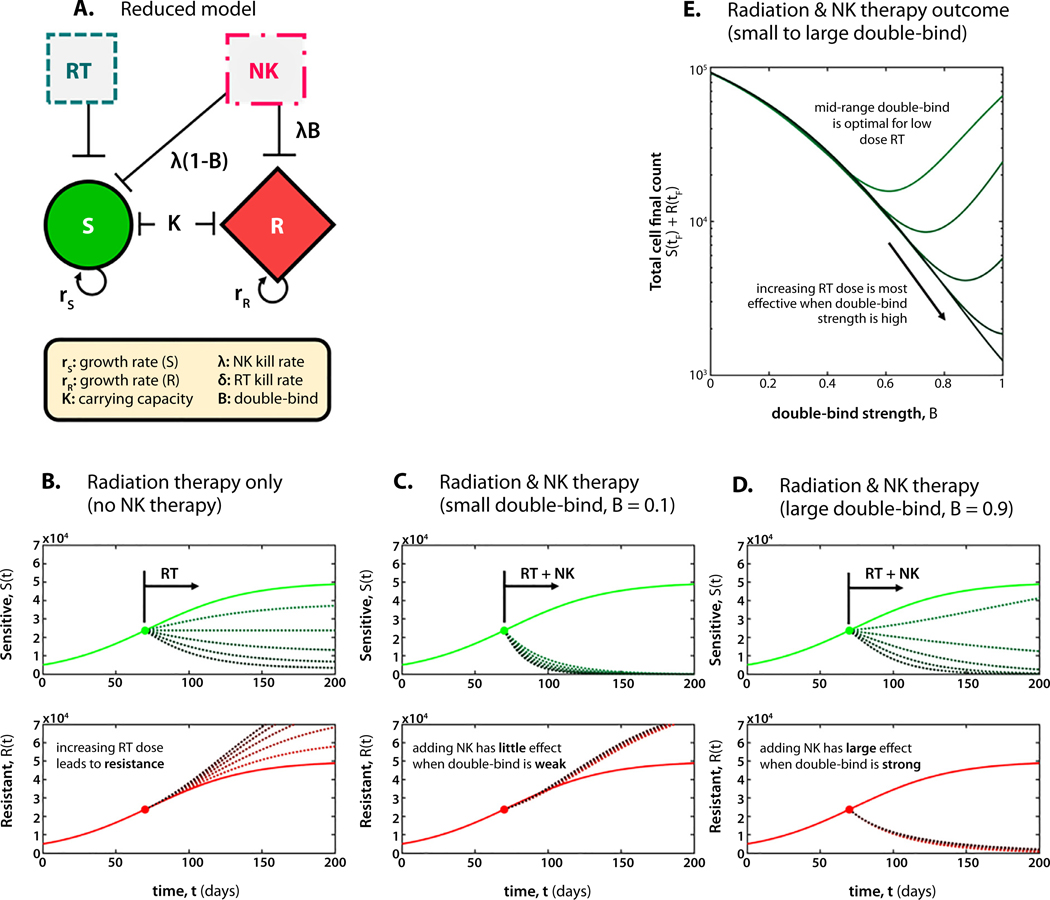
Reduced mathematical model. (A) The mathematical model is reduced to its baseline components: logistic growth, shared carrying capacity, and a double-bind parameter, as seen in Eqs. 4–6. (B) Increasing radiation therapy reduces the sensitive population (top) while selecting for resistance (bottom). Increasing values of d shown in darker colors; *δ* = [0.5, 1, 1.5, 2, 2.5]. (C) When the double-bind is weak (*B* = 0.1), the addition of NK therapy further reduces the sensitive population, but has little effect on resistance. (D) When the double-bind is strong, the addition of NK therapy reduces the resistant population, and the sensitive population can be controlled through radiation. (E) Full range of double-bind parameter, 0≤*B*≤1. Parameters unless otherwise noted: *λ* = 0.05; *K* = 1e5.

The simplified model is used to gain intuition about the role of preferential targeting by the secondary drug in an evolutionary double-bind. Figure 7 shows that an increasing dose of RT reduces the sensitive population (Fig. 7B, top) and selects for resistance (Fig. 7B, bottom). Figure 7C-E illustrates the importance of the double-bind parameter when considering a second drug as a candidate for combination therapy. When the evolutionary double-bind is weak (*B* ≈ 0), the effect of adding this second drug only serves to further reduce the sensitive population (Fig. 7C) while not fore-stalling the emergence of resistance. In contrast, when the double-bind is strong (*B* ≈ 1), the second drug preferentially targets the resistant population. In general, the final sensitive population size increases with *B*, whereas the final resistant population size decreases with *B* (Fig. 7E). Thus, a viable clinical strategy is to screen for drugs with a strong evolutionary double-bind to suppress resistance to front-line therapies, which in turn strongly target treatment-sensitive populations.

## Discussion

Evolution of resistance remains a substantial barrier to control and cure of clinical metastatic cancers by single treatments applied using the traditional oncologic strategy of continuous treatment at maximum tolerated dose until progression.^13,45–47^ Typically, second-line therapy is less effective than front-line treatment, in part because of cross-resistance to first-line therapy, which results in some protection to subsequent lines of therapy. In contrast, double-bind therapy anticipates the cancer cell’s adaptive response to an initial therapy and then specifically exploits that strategy with the follow-on treatment.^11^ Thus, the double-bind strategy is designed to *increase* the efficacy of second-line therapy. Ideally, these dynamics result in second-line treatment that is more effective than initial treatment. Importantly, therapies that target resistant strategies to front-line drugs are often less effective when given first. As a result, identifying optimal second-line therapies requires a detailed understanding of the underlying evolutionary dynamics, because their efficacy as isolated monotherapies may obscure their potential when used as part of a double-bind therapy.

Here, we used a combined empirical and theoretical investigative strategy to understand the eco-evolutionary dynamics affecting double-bind therapy optimization. Specifically, we demonstrate that radiation resistance alters NK cell ligand expression and increases sensitivity to NK cell-mediated killing in an isogenic cell line model. Radiation induces activation of double-stranded DNA break repair pathways, homologous recombination, and nonhomologous DNA end joining.^37,48–51^ Increased expression of repair pathway proteins (DNA-PK, ATM, Rad52, MLH1, and BRCA1) is associated with cancer cell survival following radiation.^37,52^ Therefore, NK cell-based immunotherapies are an obvious choice for combination with DNA-damaging agents for a double-bind strategy. Radionuclide therapy targeting PSMA^+^ metastatic PC delivers localized radiation to metastatic lesions inducing DNA damage.^53,54^ We are currently exploring this double-bind in metastatic hormone-resistant PC. Here, we focus on sequential therapy using RT followed by NK cells to test the hypothesis that NK cells can preferentially target tumor cells that have developed resistance to RT therapy. However, NK cells given before RT may also be beneficial if given with consideration for the complex effect of RT on NK cell viability and function.^55^ We are currently including this in ongoing studies.

The interaction dynamics between the tumor and components of the immune system are difficult to study. In vitro models are limited by space and lack temporal information as well as ecological context. *In vivo* models rely on humanized mouse models or the murine immune system. Immunologists are increasingly using computational modeling to help fill the gaps in experimental systems and expedite bench-to-bedside translation.^56,57^ To our knowledge, this manuscript provides the first direct experimental evidence quantifying a treatment double-bind in PC. Our work is also mathematically novel in that it extends EGT models to consider the competitive effect of NK cells on treatment-naïve and RR lines, thus adding a new dynamic to the double-bind concept: preferential targeting of RT-resistance. These mathematical models can also be used with other in vitro model systems and can be extended to include additional therapy considerations such as dose, timing, and additional treatment modalities.

Combination therapy is highly dependent on drug selection. Combining treatments with complementary mechanisms of action can increase responses by targeting different cellular pathways. Similarly, collateral sensitivity occurs when the application of 1 drug increases the effects of a second drug and is identified when a second drug functions better in a resistant line than in the parental line.^58,59^ An evolutionary double-bind builds on these vulnerabilities by considering the population structure including fitness trade-offs and ecological interactions. Targeting multiple pathways and identifying collateral sensitivities can enable a double-bind; however, an evolutionary double-bind requires competition dynamics and uses drug sequencing to leverage fitness trade-offs.

The theoretical literature often assumes a priori a fitness cost of adaptive resistance mechanisms.^8,60^ Here, we investigated both an intrinsic fitness cost to growth rates and a frequency-dependent fitness advantage and found neither. In fact, we observed a growth rate benefit to resistant cells, albeit at a cost to their carrying capacity, and suppression (via competitive exclusion) of sensitive cells, which is rare but not unprecedented.^61^ This is reminiscent of the *r*/*K* selection theory described in the literature of both antibiotics^62,63^ and cancer,^64,65^ which there lies an evolutionary trade-off between maximizing fitness through increased proliferation (*r*) or carrying capacity (*K*). Previously proposed evolution-based treatment strategies, such as adaptive therapy, have shown that a cost of resistance is a necessary prerequisite.^60,66,67^ In contrast, the evolutionary double-bind treatment strategy of NK cell-based therapy represents a promising alternative that neither requires a cost of resistance nor dose modulation but rather focuses on the critical order of treatment. It should be noted that 1 limitation of the model is the lack of an interaction term in which tumor cells interact with NK cells, as it is likely that there exists a feedback whereby tumor cells stimulate the growth of NK cells.

We have demonstrated an effective double-bind therapy using RT and NK cells in vitro. These findings will need to be tested in additional model systems that include more complex environmental and immunological contexts before clinical relevance can be determined. Similar to in vitro models used to identify synergistic effects of combination therapies, “gold-standard” experimental procedures will need to be defined for high-throughput identification of double-bind combination therapies. Our findings provide an experimental setup that highlights the need for mixed cultures in double-bind studies. Preclinical screening for additional double binds would require testing combination therapy in which the first drug has known resistance mechanisms that can be targeted by a follow-on therapy using assays that allow for competition among cell types and can be quantified with a mathematical model.

## Methods and Materials

### Cell lines

DU145 and PC3 cells were derived from in-house stocks. Parental 22Rv1 and the isogenic cell lines AMC-22Rv1 and RR-22Rv1 were previously derived from parental line 22Rv1 with either fractionated radiation (30 × 2 Gy) or mock irradiation.^37^ The resulting cell lines, radiation-resistant (RR-22Rv1) and age-matched control (AMC-22Rv1), have differential sensitivity to x-ray radiation. Cells were maintained in RPMI (Gibco) with 10% fetal bovine serum (FBS) and 1% pen/step in a humidified incubator at 37°C with 5% CO_2_. Cells were kept in log phase unless specified. NKL cells (an immortalized NK cell line) were obtained from internal lab stocks and cultured in RPMI with 10% FBS, 1% pen/strep, and 100 IU recombinant human IL-2. Cell lines were authenticated, and mycoplasma tested regularly (Lonza).

Stable GFP and mCherry expressing cell lines were created through lentiviral transduction with pLV(Exp)-CAG>3xNLS/EGFP-P2-A-puro and pLV(Exp)-CAG>3xNLS/mCherry-P2-A-puro, respectively. Lentivirus-EGFP and -mCherry were kindly provided by the Marusyk Lab. Positively transduced cells were selected with 2 *μ*g/mL puromycin, followed by a sterile sort for high GFP and mCherry expression (BD FACSAria II). Expression was maintained with 1 *μ*g/mL puromycin in all experiments except those involving immune cells.

### Flow cytometry

Following treatment (where applicable), tumor cell lines were stained with Zombie Aqua Fixable viability dye (1:1000 in PBS) and for NK ligand (BioLegend) expression in PBS containing 5% FBS and 0.1% sodium azide for 20–30 minutes at room temperature. Secondary antibody was added for ULBP 1 and 2/5/6 only (R&D Systems). Cells were then washed and fixed in 4% paraformaldehyde. Flow was performed on BD LSR II and analyzed using FlowJo software (Tree Star). Internal controls were used in all experiments, and technical and biological replicates were always performed in the same facility to remove intermachine variability. Morphology gating, viability gating, and doublet discriminator gates were used prior to calculating MFI. Mean fluorescence intensity was determined as MFI stained-MFI unstained. ULBP primary and secondary antibodies were purchased from R&D Systems; all others were preconjugated and obtained from BioLegend.

### Radiation

In vitro radiation was given in either 2, 6, 10, or 15 Gy doses using the XRAD 160 biological irradiator (Precision X-Ray Inc). Prior to RT, cells were plated in complete media with 10% FBS and 5% pen/strep and left to adhere overnight. The following RT cells were immediately returned to the incubator at 37 °C with 5% CO_2_.

### Competition experiments with IncuCyte imager

Cells were counted and mixed at various ratios prior to plating in 96-well flat-bottom plates (Corning). Tumor cells were plated at 2000 cells per well and left to adhere. Images were taken every 6 hours for 150 hours on the IncuCyte ZOOM. Cell number was determined by the IncuCyte software. Three technical replicates were used for each run. Normalized cell counts were calculated to allow for growth rate comparison between the different ratios (time *t*/time 0).

### Statistical analysis

Prism (version 9, GraphPad software) was used for all statistical analyses. A Student *t* test, one-way ANOVA with Bonferroni correction, and two-way ANOVA were used when the means of more than 2 groups were compared. All experiments were performed with 3 technical replicates. A minimum of 10,000 events was collected for each flow sample. n.s. = not significant, *P < .05, **P < .01, ***P < .001, and ****P < .0001.

### Mathematical model development

Below, we introduce a two-population LV competition model, consisting of sensitive (*S*) and radiation-resistant (*R*). Both cell types compete for space and resources according to a fixed carrying capacity, *K*_*i*_, and a cell-type-specific growth rate, *r*_*i*_, in which *i* = [*S,R,N*]. The inter-specific competition terms are given by ⍺_ij_, describing the competition of cell type *j* on cell type *i*.


(1) $$\frac{dS}{dt}=r_{S}S\left(1-\frac{S+\alpha_{SR}R}{K_{S}}\right)-\lambda_{S}SN$$

(2) $$\frac{dR}{dt}=r_{R}R\left(1-\frac{R+\alpha_{RS}S}{K_{R}}\right)-\lambda_{R}RN$$

In our experimental system, we make the simplifying assumption that NK cells grow with a logistic growth dynamic, with no competitive feedback from *S* or *R* cells:

(3) $$\frac{dN}{dt}=r_{N}N\left(1-\frac{N}{K_{N}}\right)$$

### Fitting process

The following procedure was used when fitting longitudinal experimental data. The dataset contains cell counts for sensitive, *S*(*t*), and radiation-resistant, *R*(*t*), cells over time every 6 hours over approximately 150 hours, repeated for 12 replicates. Imaging and fluorescence do not give any information about *N*(*t*), so we set *K*_*N*_ = 1 × 10^5^. Note that the sign of the competition parameters (*λ*_*S*_, *λ*_*R*_, *α*_SR_, and *α*_RS_) is not affected by this choice of *K*_*N*_, as the state-space of *N*(*t*) in Eq. 3 is always nonnegative. Let *f* be the initial fraction of radiation-resistant cells in the experimental setup such that a “fitting batch” represents a single replicate for *f* = [0, 0.10, 0.25, 0.50, 0.75, 0.90, 1]. Data for this single replicate are fit to the model simultaneously across all values of *f* and subsequently repeated for each of the 12 replicates. In this way, each parameter (*r*_*S*_*, r*_*R*_, …) has a mean value and standard deviation.

Fitting is done for the following: (1) untreated, (2) radiation treatment only, (3) NK cells only, and (4) combination. Due to concerns about the identifiability of the carrying capacity in the treated experimental conditions, the untreated condition is fit first, and all other treated experimental conditions are fit using the mean value for carrying capacities derived in the untreated condition (*K*_*S*_ and *K*_*R*_). Growth rates (*r*_*i*_) are bound between 0 and 1, competition values (*λ*_ij_) are bound between −15 and 15, and carrying capacities (*K*_*i*_) are bound between 0 and 10^6^. For experimental setups 1 and 2 with no NK cells, the effect of NK cells on *S* and *R* is set to zero: *λ*_*S*_ = *λ*_*R*_ = 0. A validation of the robustness of the parameter fitting process of the mathematical model is shown in Figure E6 by splitting the data into a testing and validation set.

### Reduced model

In Figure 7, the mathematical model is reduced to its baseline components: intrinsic growth rates, *r*_*S*_ and *r*_*R*_, shared carrying capacity, *K*, RT kill rate, *δ*, NK cell kill rate, *λ*, and a double-bind parameter, *B*. RT reduces the growth rate of sensitive cells only by a fraction *δ*. NK cells kill cancer cells at a rate *λ*, in which the double-bind parameter alters the second drug’s kill effect to target only sensitive cells (*B* = 0), target only resistant cells (*B* = 1), or a given ratio of sensitive and resistant cells (0 < *B* < 1). A cost of resistance reduces the growth rate of resistant cells by a fraction *c*.

(4) $$\frac{dS}{dt}=r_{S}S\left(1-\frac{S+R}{K}\right)-\lambda S(1-B)$$

(5) $$\frac{dR}{dt}=r_{R}R\left(1-\frac{R+S}{K}\right)-\lambda SB$$

where RT reduces the growth rate of sensitive cells during therapy by a fraction of (1-δ):

(6) $$\overset{´}{r_{S}}=r_{S}(1-\delta )$$

or rewriting to solve for δ:

(7) $$\delta =1-\frac{\overset{´}{r_{S}}}{r_{S}}$$

## Supplementary Material

2

3

4

5

6

7

Supplementary material associated with this article can be found in the online version at doi:10.1016/j.ijrobp.2025.09.034.

## Figures and Tables

**Table 1:** Parameter estimates under different treatment conditions

	*r_s_* Growth rate (*S*)	*r_R_* Growth rate (*R*)	*r_N_* Growth rate (*N*)	*K_s_* Carrying capacity (*S*)	*K_R_* Carrying capacity (*R*)	*K_N_* Carrying capacity (*N*)	*α*_SR_ Competition effect of *R* on *S*	*α*_RS_ Competition effect of *S* on *R*	*λ*_SN_ NK kill rate of S cells	*λ*_RN_ NK kill rate of R cells
UT	0.032 (0.0315–0.0325)	0.0357 (0.035–0.0363)	**0** (fixed)	1.27 (1.24–1.31)	0.779 (0.757–0.8)	**1** (fixed)	1.54 (1.45–1.63)	0.3 (0.254–0.347)	**0** (fixed)	**0** (fixed)
NK	0.034 (0.033–0.0349)	0.0438 (0.0425–0.045)	0.0178 (0.0162–0.0193)	**1.27** (fixed)	**0.779** (fixed)	**1** (fixed)	1.38 (1.3–1.46)	0.0526 (−0.0334 to 0.139)	0.0308 (0.0242–0.0374)	0.0558 (0.0471–0.0645)
RT	0.0162 (0.0158–0.0167)	0.0218 (0.0211–0.0225)	**0** (fixed)	**1.27** (fixed)	**0.779** (fixed)	**1** (fixed)	2.36 (1.79–2.93)	0.99 (0.68–1.3)	**0** (fixed)	**0** (fixed)
RT+NK	0.023 (0.0199–0.0262)	0.0331 (0.0268–0.0394)	0.0245 (0.0204–0.0286)	**1.27** (fixed)	**0.779** (fixed)	**1** (fixed)	1.96 (1.33–2.59)	1.83 (0.809–2.85)	0.0865 (0.0488–0.124)	0.0945 (0.0545–0.135)

Mean values across 12 replicates with plus or minus 1 standard error measurement shown in parentheses. See Eqs. 1–3.

Bold face parameters are fixed during the fitting process.

## Data Availability

Code used to produce the figures will be available on our department’s GitHub account: https://github.com/MathOnco/RT-NK-evolutionary-double-bind.
