# Supplementary figures and images for: Evolutionary Double-Bind Treatment Using Radiation Therapy and Natural Killer Cell-Based Immunotherapy in Prostate Cancer
Source: Int J Radiat Oncol Biol Phys. Author manuscript; Available in PMC 2026 Feb 16. (PMC12908384; doi:10.1016/j.ijrobp.2025.09.034)

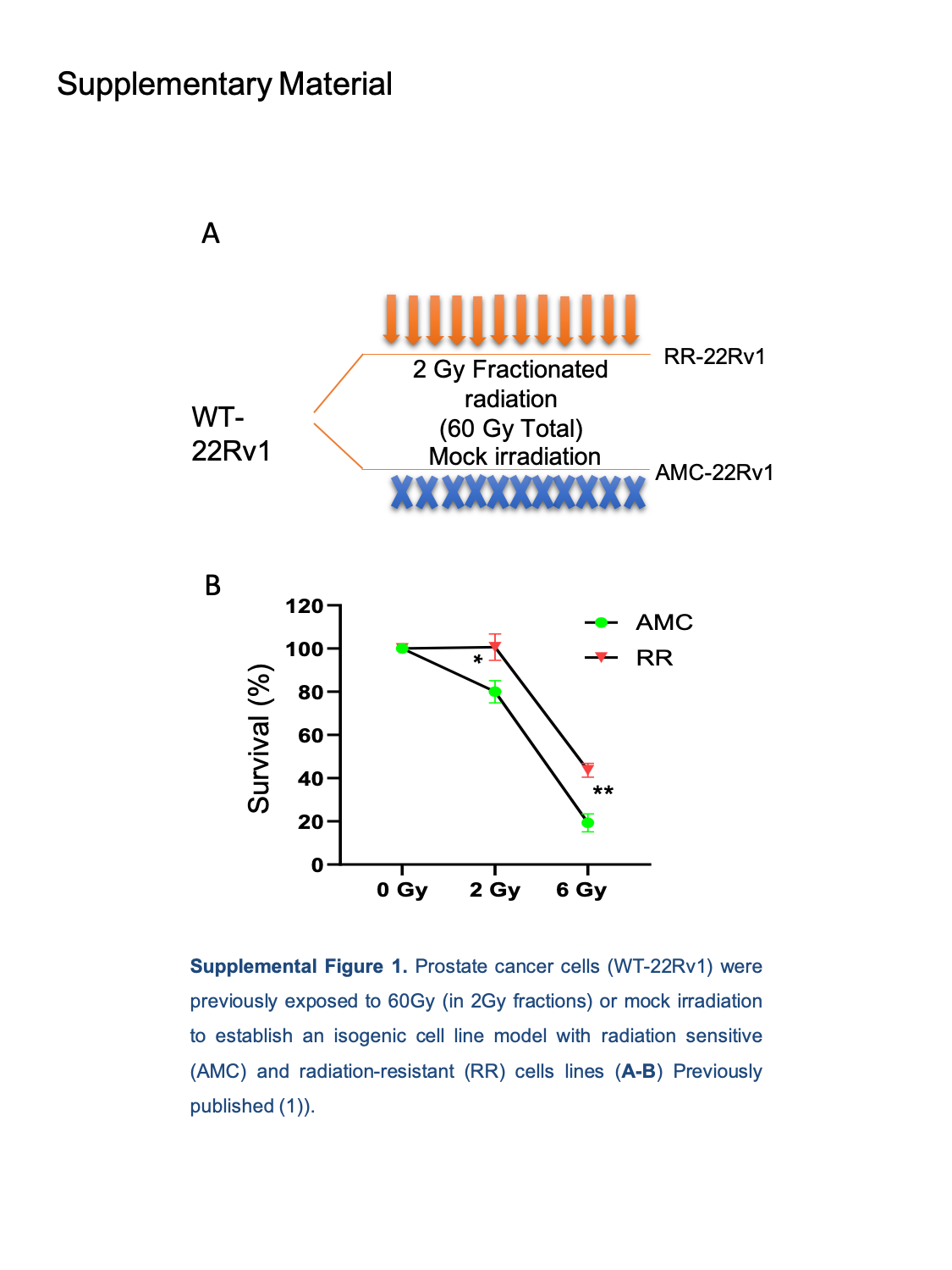

Supplement: 2 [file NIHMS2119323-supplement-2.png]

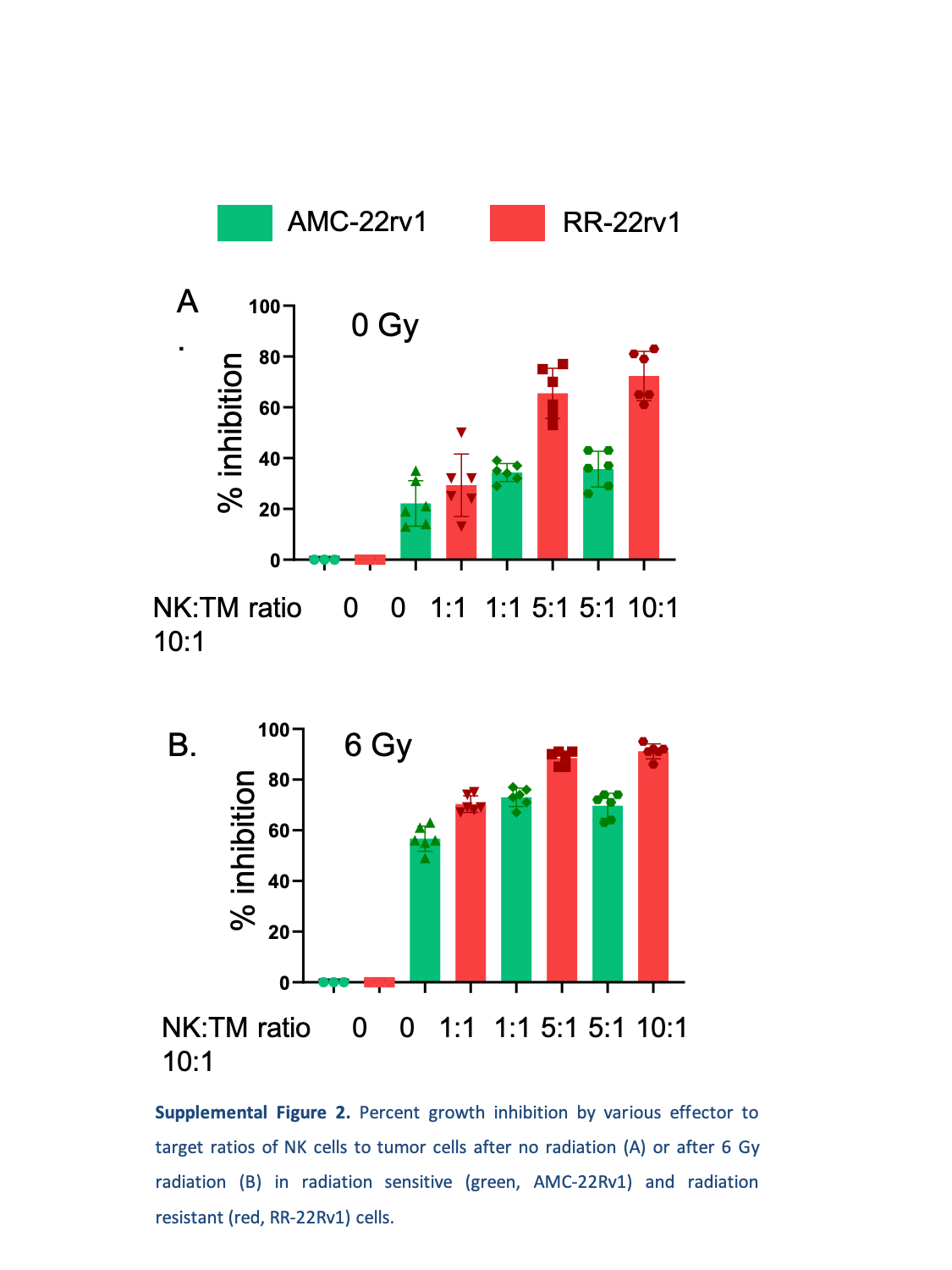

Supplement: 3 [file NIHMS2119323-supplement-3.png]

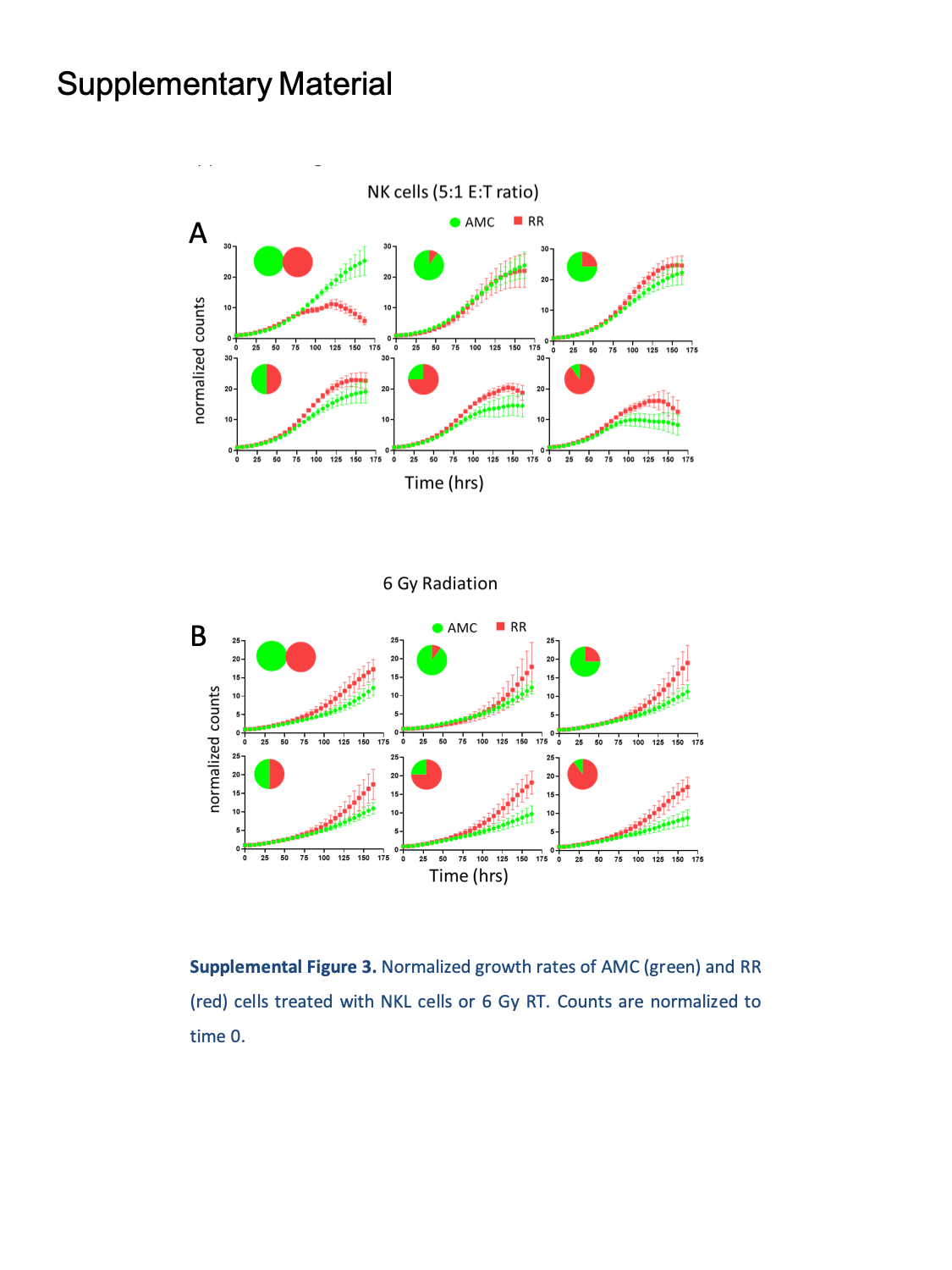

Supplement: 4 [file NIHMS2119323-supplement-4.png]

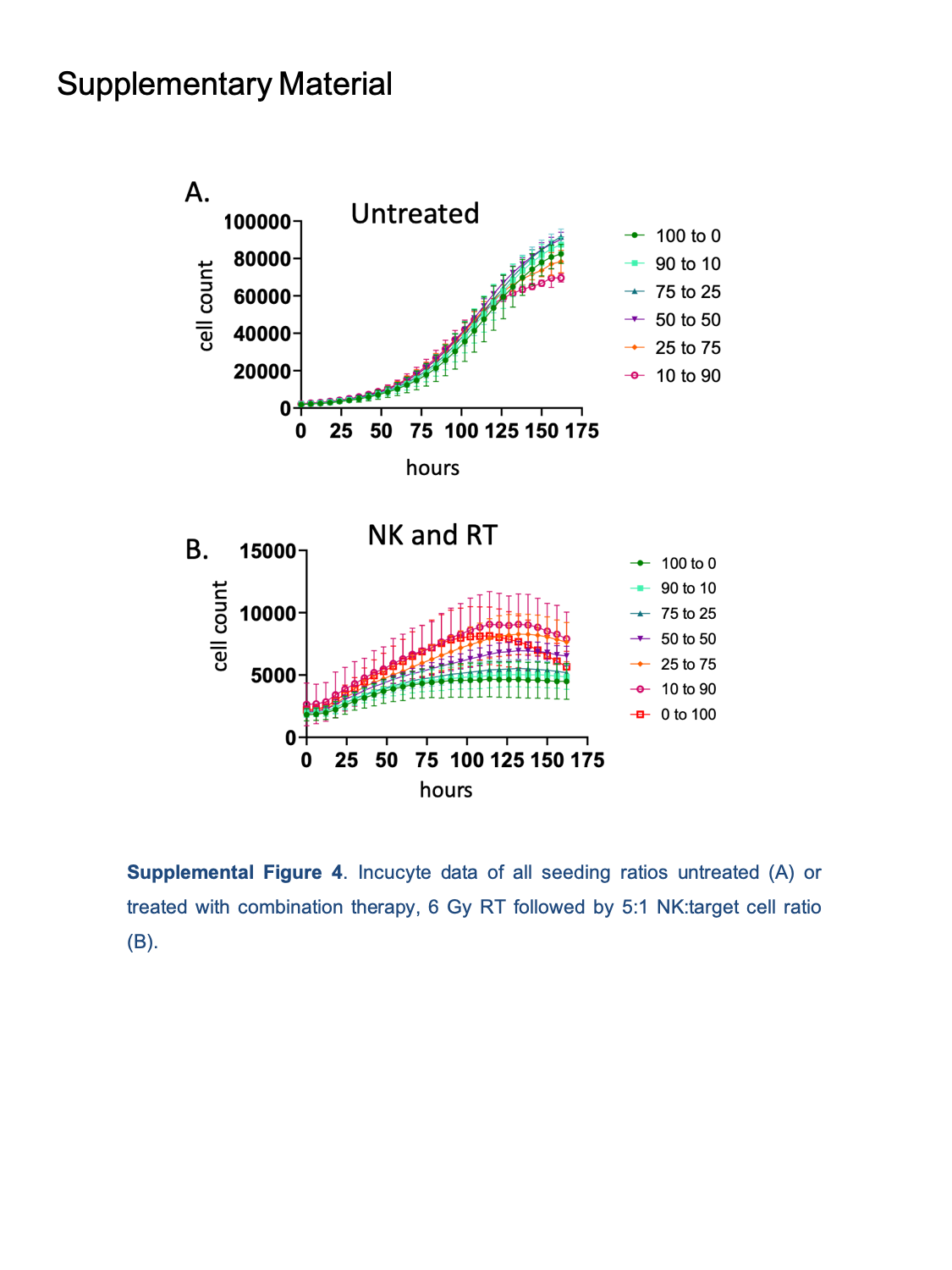

Supplement: 5 [file NIHMS2119323-supplement-5.png]

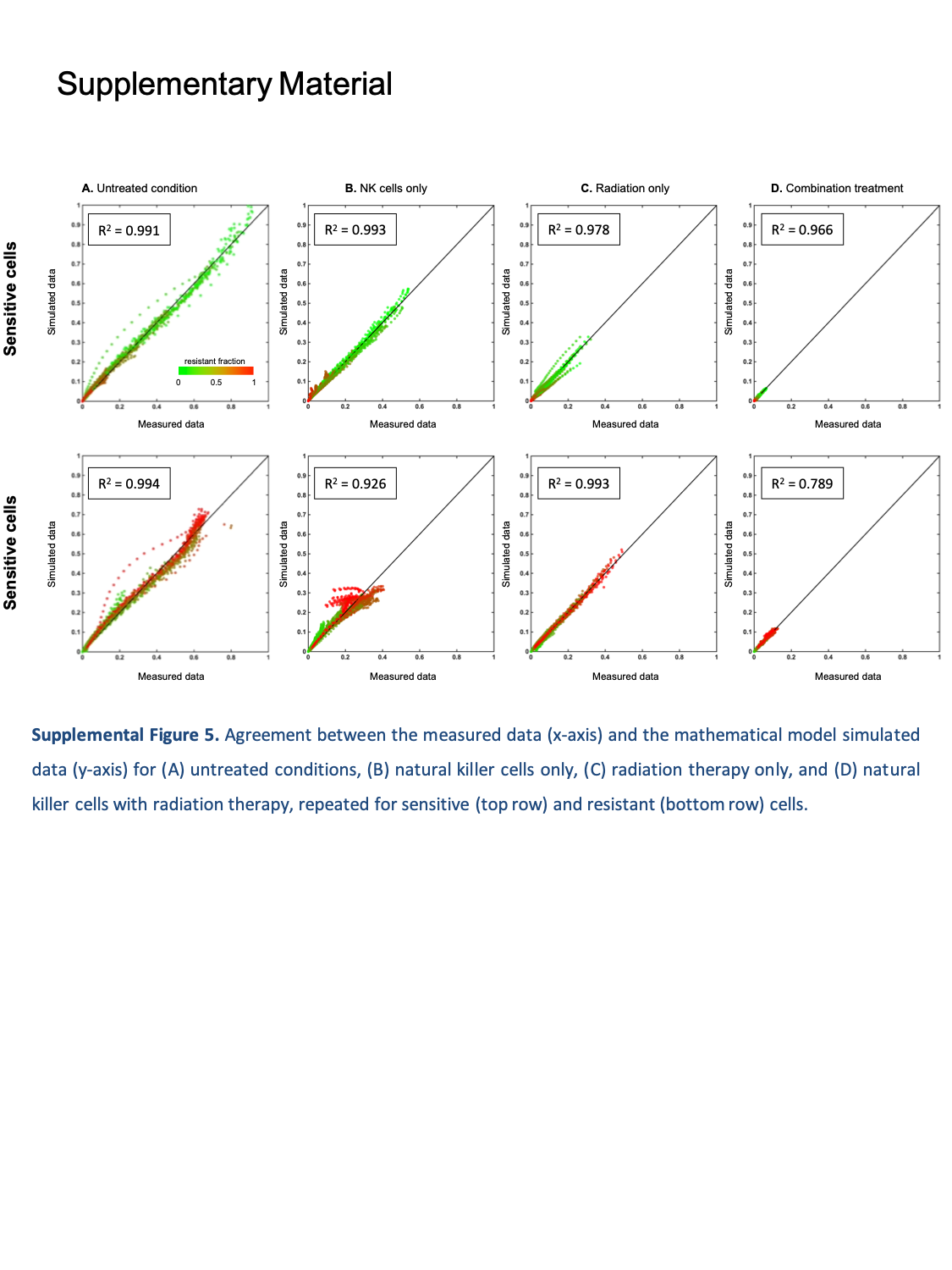

Supplement: 6 [file NIHMS2119323-supplement-6.png]

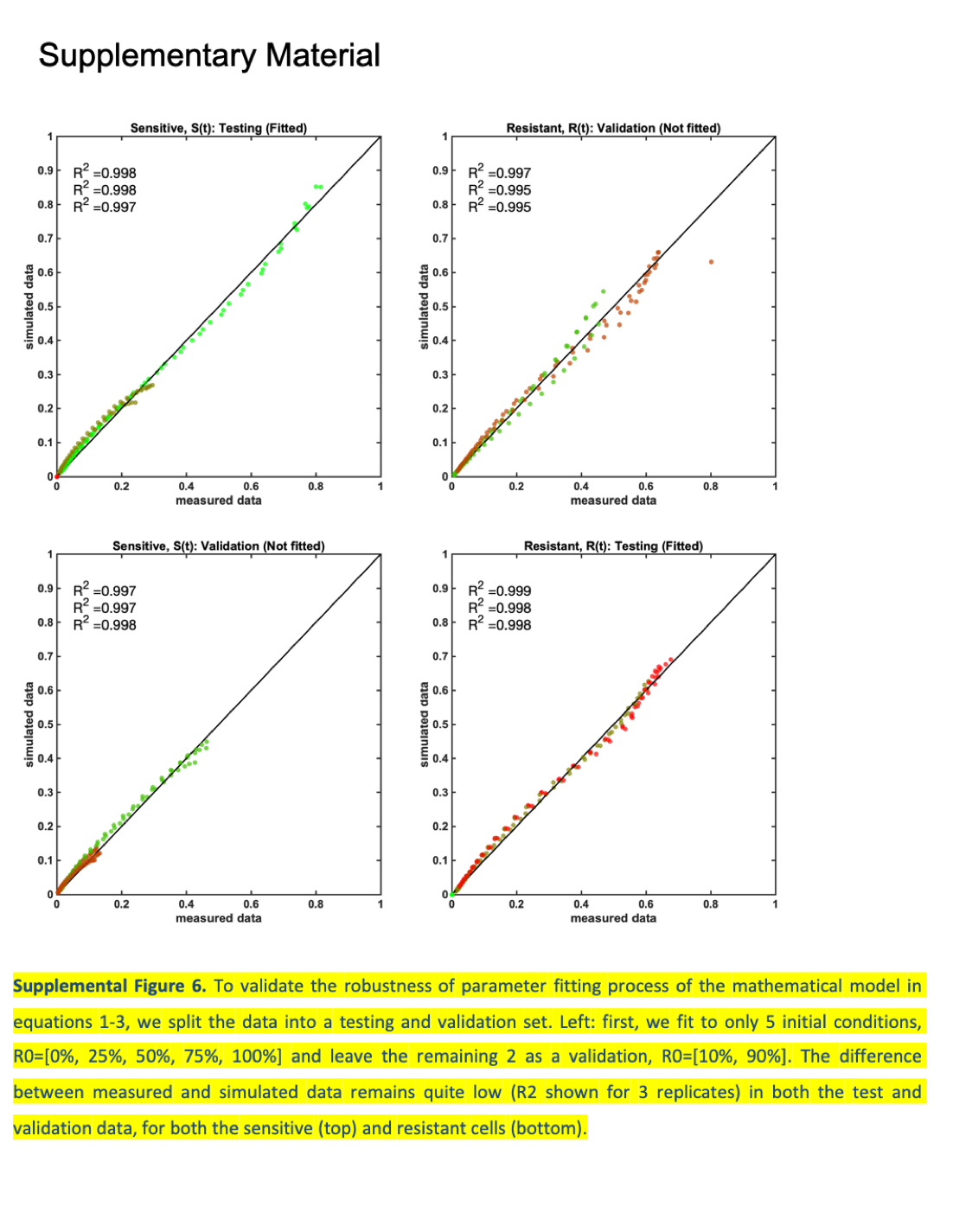

Supplement: 7 [file NIHMS2119323-supplement-7.png]
